# Isolation of methyl gamma linolenate from spirulina platensis using flash chromatography and its apoptosis inducing effect

**DOI:** 10.1186/s12906-015-0771-8

**Published:** 2015-08-04

**Authors:** Jubie S., Dhanabal S. P., Chaitanya M. V. N. L.

**Affiliations:** Department of Pharmaceutical chemistry, JSS College of Pharmacy, JSS University, Mysore, India; Department of Pharmacognosy and Phytopharmacy, J. S. S. College of Pharmacy, Rock lands, Ooty-643 001 Tamilnadu, India; Research Associate, Department of Pharmacognosy and Phytopharmacy, JSS College of Pharmacy, Ooty-643001 JSS University, Mysore, India

**Keywords:** *Spirulina platensis*, Lung cancer, Gamma linolenic acid (GLA), Cytotoxicity, Flash chromatography

## Abstract

**Background:**

Isolation of methyl gamma linolenate from *Spirulina platensis* using flash chromatography and its apoptosis inducing effect against human lung carcinoma A- 549 cell lines.

**Methods:**

Gamma linolenic acid is an important omega-6 polyunsaturated fatty acid (PUFA) of medicinal interest was isolated from microalgae *Spirulina platensis* using flash chromatography system (Isolera system) as its methyl ester. The isolated methyl gamma linolenate was characterized by IR, ^1^H NMR, ^13^C NMR and mass spectral analysis and the data were consistent with the structure.

**Results:**

The percentage yield of isolated methyl gamma linolenate is found to be 71 % w/w, which is a very good yield in comparison to other conventional methods. It was subjected to i*n-vitro* cytotoxic screening on A-549 lung cancer cell lines using SRB assay and result was compared with standard rutin.

**Conclusion:**

It may be concluded that the Flash chromatography system plays a major role in improving the yield for theisolation of methyl gamma linoleate from *Spirulina platensis* and the isolated molecule is a potent cytotoxicagent towards human lung carcinoma cell lines, however it may be further taken up for an extensive study.

## Background

Spirulina is a commercial well known micro algae, which contains various antioxidants, especially phycocyanin. Apart from being sold as a nutraceutical, Spirulina is incorporated as a functional ingredient in food products and beverages. Spirulina refers to the dried biomass of *Arthrospira platensis*, belonging to the family *Phormidiaceae*, an oxygenic photosynthetic bacterium found worldwide in fresh and marine waters [1]. This algae represents an important staple diet in humans and has been used as a source of protein and vitamin supplement in humans without any significant side-effects. Apart from the high (up to 70 %) content of protein, it also contains vitamins, especially B_12_ and provitamin A (β-carotenes) and minerals especially iron. It is also rich in phenolic acids, tocopherols and gamma linolenic acid (GLA) [2, 3].

Many commercial methods are available for producing gamma linolenic acid (GLA) concentrates include winterization, fractional distillation, urea-inclusion, high performance liquid chromatography and argentated silica gel chromatography [4–7]. Even though GLA concentrates mentioned above are sufficient for most applications; however pharmaceutical applications of GLA require higher concentrations of GLA, often in excess of 90 % but many of these methods did not produce the satisfied output [8]. Hence new method for producing higher concentrations of gamma linolenic acid and its ester forms from *Spirulina platensis* are urgently needed. Lung cancer is one of the leading causes of cancer deaths (15 %) in the world and is by far the most common cancer in the western world. Globally lung cancer is responsible for more deaths annually than those due to breast cancer, colorectal cancer, and prostate cancer combined together [9–11]. As per the National Institute of Health Analysis (NIH) report, the medical expenditure for the diagnosis and treatment of cancer could be as high as $ 207 billion, out of which $ 12 billion could be only for the treatment of lung cancer [12].

A number of investigations have demonstrated that, a variety of modified fatty acid analogues are promising molecules in cancer prevention and have potential in the treatment of cancer [13, 14]. Some studies have shown that GLA can slow or stop the growth of some types of cancer cells in tissue cultures in the laboratory. The same kinds of studies suggest that GLA may help some cancer drugs to work better [15–18]. However, there is very little evidence as yet that GLA work to prevent or treat cancers in humans. Human studies are under way to evaluate the role of GLA and other essential fatty acids on the growth of cancer. As existing anticancer drugs of synthetic origin are having more adverse effects, the developments of safe compounds are urgently needed. In recent years, more importance has been given to nutraceuticals/functional foods because they can provide physiological benefits additional to nutritional and energetic, as for instance antihypertensive and anticancer activities. Since the gamma linolenic acid has come under the category of Nutraceuticals/functional foods, its exploration in the treatment of lung cancer may be considered as safe. Considering all these facts, the present research is aimed to produce the high concentrates of methyl gamma linolenate from *Spirulina platensis* using flash chromatography as a molecular target towards treatment of lung cancer and the isolated product is characterized by using HPTLC, NMR and mass spectra. The characterized molecule is subjected to *in*-*vitro* cytotoxic studies on human lung carcinoma A-549 cell lines.

## Methods

### General

All chemicals used were purchased from Fluka chemicals. Their purity was checked by GC. All solvents were purified by distillation using Rotavap (Buchi R120) and if necessary residual water was removed. The components of solvents and elements are given in volume ratios of the components. Methyl gamma linolenate was isolated and purified by using Isolera Flash chromatography System (Biotage INC) and identified using different spectral and HPTLC techniques. The standard methyl gamma linolenate and routine were purchased from Sigma-Aldrich uses thin layer chromatography. The ^1^H NMR and ^13^C NMR were recorded on Bruker DRX-300 (300 MHz FT-NMR) using deuterated chloroform as solvent and TMS as internal standard. The mass spectra of compounds were recorded on JEOL GCMATE II GC-MS.

### Algae

Fresh cultures of *Spirulina platensis* were obtained from Antenna Research Foundation Pvt Ltd., “Madurai, Tamilnadu, India”. The cell paste was lyophilized and stored at −20 °C for further use.

### General procedure for isolation of methyl gamma linolenate

The Isolera system is a high-performance flash chromatography system, consisting of the following integrated components:

#### Isolera flash chromatography system

A touch screen display, which is a solvent-resistant, color LCD screen with a resolution of 800 × 600 pixels. It serves both as a display and as the system’s input device via on-screen touch controls. A fraction collector, which collects fractions into a wide variety of collection racks and vessels. A pump module, which directs liquid flow through the system. A default flow rate is specified for each cartridge but, if desired, the flow rate can be changed. If the flow rate is increased, the system will start the run at the default flow rate and then regulate towards the flow rate defined in the method. The system regulates on both flow rate and pressure. If 90 % of the maximum allowed pressure is reached before the defined flow rate, the flow rate at 90 % pressure will be used. An internal detector, which provides the system with information on the light absorbance of the solvents and samples passing through the detector flow cell. The different fractions can be collected through a automated fraction collector based on the R_f_ the compound of interest can be identified and pooled together after performing thin layer chromatography analysis.

#### Preparation of fatty acid methyl esters (FAME)

Freeze dried biomass of *Spirulina platensis* (50 g) was extracted by reflux for 4 h using a mixture of methanol and acetyl chloride (95:5, 800 ml). The extract obtained was diluted with water and extracted thrice with equal volume of n-hexane containing 0.01 % butylated hydroxyl toluene. The combined n-hexane layer was evaporated to get the fatty acid methyl ester (FAME).

#### Enrichment of FAME for methyl gamma linolenate (GLA-ME)

The FAME obtained was subjected to urea complexation as described previously to remove the saturated fatty acids from the polyunsaturated fatty acids. For the urea complexation, methanol (9 ml) and urea (3 g) were added to FAME. The mixture was heated to get a clear solution. It was cooled at room temperature and stored at 0 °C overnight. Then it was filtered to remove the crystals settled at the bottom. The filtrate was extracted with n- hexane containing 0.01 % butylated hydroxyl toluene. The n-hexane fraction was evaluated for the presence of GLA methyl ester by HPTLC.

#### Quantification of GLA-ME in enriched GLA fraction by HPTLC

##### Preparation of sample solution

Whole enriched GLA-ME fraction (122.5 mg) was dissolved in 10 ml of n-hexane. This solution was used for application on to the TLC plate.

##### Preparation of standard solution

Standard GLA-ME was prepared by dissolving 27 mg of GLA-ME and in 10 ml of methanol in a volumetric flask.

##### Chromatographic conditions

Stationary phase : Silica gel 60 F 254

Mobile phase : Hexane: Acetone (6:4)

Mobile phase volume : 10 ml

Band length : 6 mm

Application rate : 10 s/μl

Development chamber : Camag twin trough development chamber (10 × 10 cm)

Development distance : 6 cm from the application position

Scanner : Camag TLC scanner III

Detection wavelength : 235 nm

Slit dimension : 4.00 × 0.30 mm

Scanning speed : 20 mm/s

Data resolution : 100 μm/step

Measurement mode : Absorption

Peak area of GLA-ME standard = 1379.2

Peak area of GLA-ME in sample = 3605.6

The amount of GLA-ME was quantified by the following formula;

$$ \begin{array}{l}\%\ \mathrm{of}\ \mathrm{G}\mathrm{L}\mathrm{A}\hbox{-} \mathrm{ME}=\kern1em \frac{\mathrm{Sample}\ \mathrm{peak}\ \mathrm{area} \times \mathrm{s}\mathrm{t}\mathrm{d}.\ \mathrm{weight} \times \mathrm{vol}.\ \mathrm{d}\mathrm{i}\mathrm{l} \times \mathrm{s}\mathrm{t}\mathrm{d}.\ \mathrm{applied}}{\mathrm{Std}\ \mathrm{peak}\ \mathrm{area} \times \mathrm{vol}.\ \mathrm{applied} \times \mathrm{s}\mathrm{ample}\ \mathrm{weight} \times \mathrm{s}\mathrm{ample}\ \mathrm{applied}} \times 100\hfill \\ {}\hfill \\ {}\hfill \end{array} $$

#### Separation of GLA-ME using flash chromatography system

**Fig. 1:**
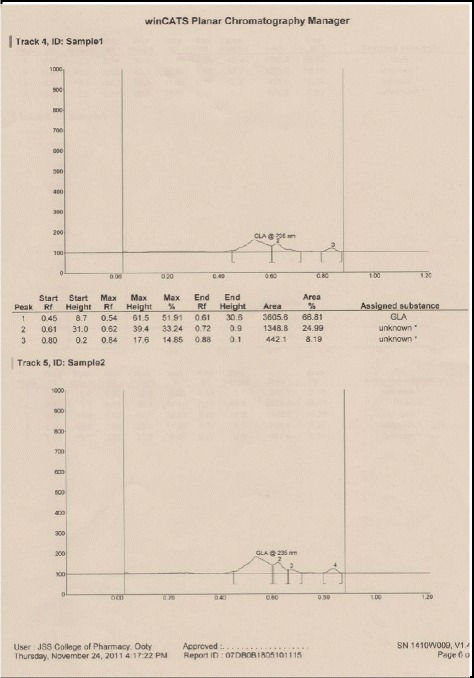
HPTLC Chromatogram of GLA- ME

**Fig. 2:**
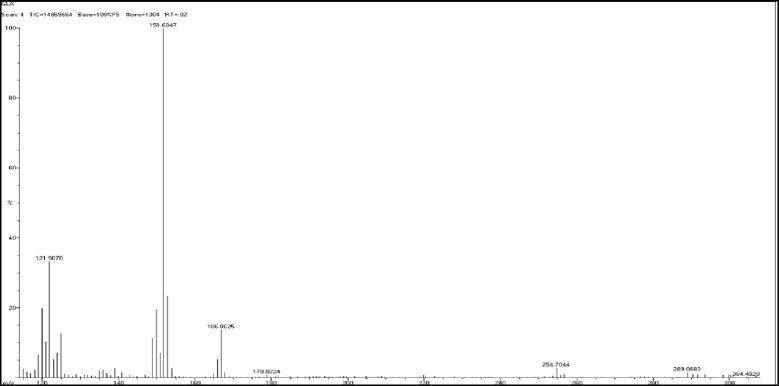
Mass spectra of GLA-ME

**Fig. 3:**
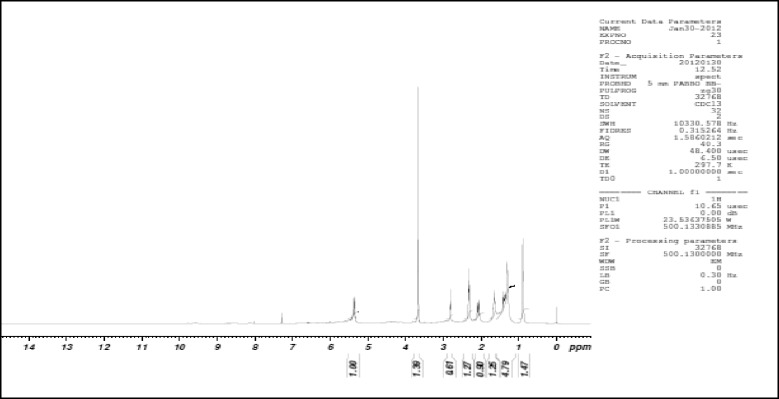
^1^H NMR of GLA-ME

**Fig. 4:**
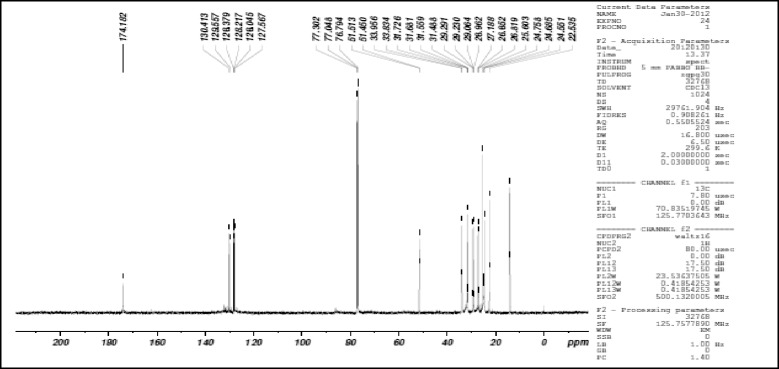
^13^C NMR of GLA-ME

**Fig. 5:**
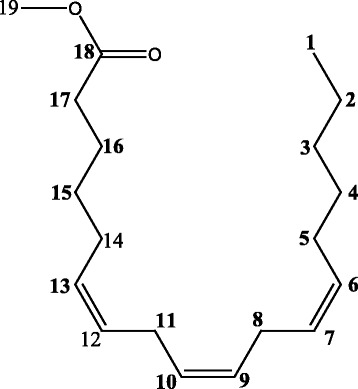
GLA-ME

**Fig. 6:**
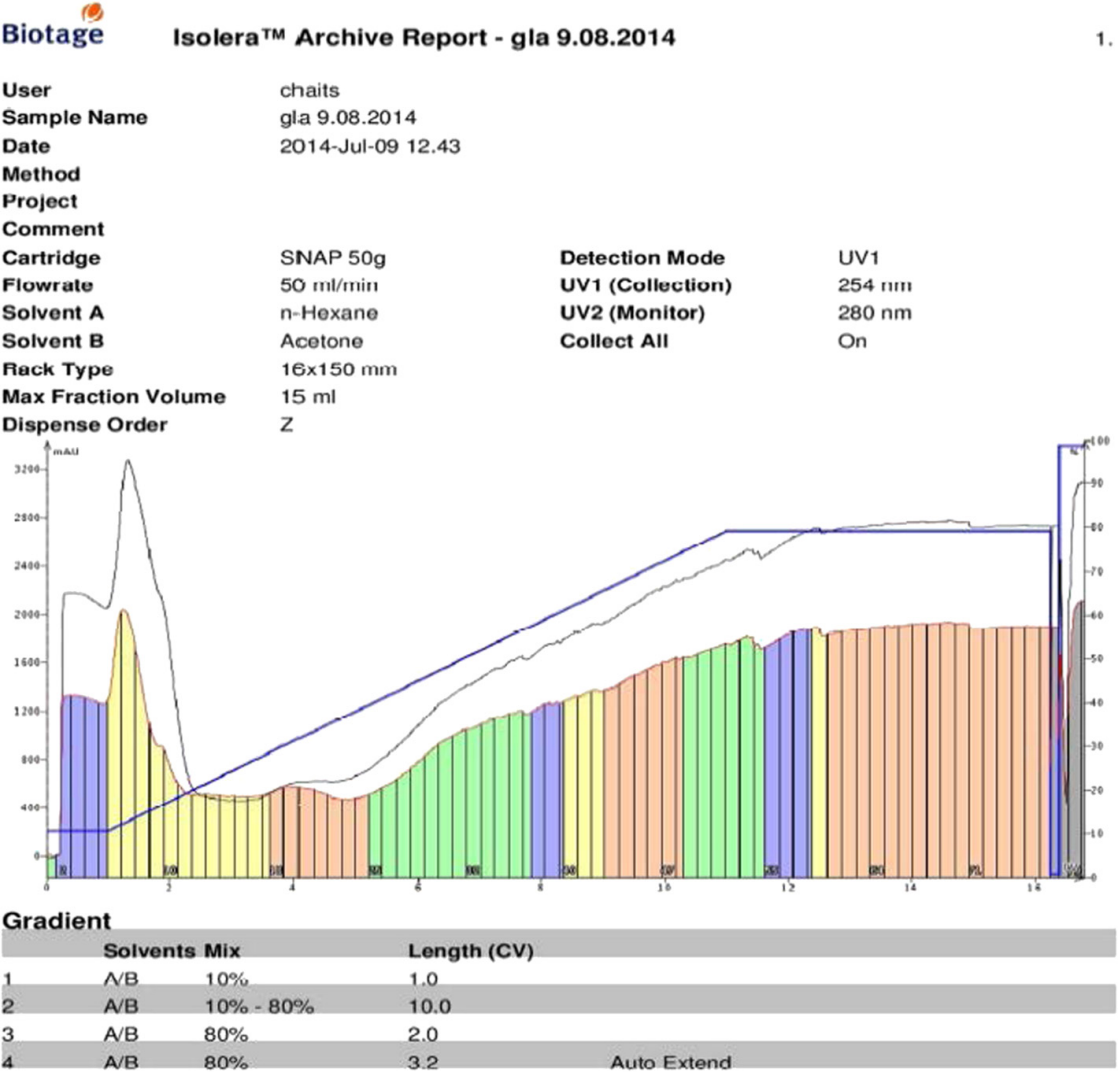
Flash Chromatogram of GLA- ME ISOLATION

0.5 g of enriched GLA fraction obtained above was directly applied on 10 g samplet and the samplet was dried under vacuum in a rotary evaporator (Buchi R 120). The dried samplet was packed in 50 g KPSil Biotage SNAP Cartridge. A flash chromatography method was developed based on HPTLC profile. A constant flow rate 50 ml/min of mobile phase (60 % hexane: 40 % acetone) is used. A total no of 105 fractions, each 22 ml was collected in different test tubes at the wavelength of 254 nm. Each individual fraction was subjected to TLC Analysis using the above mobile phase in comparison with that of standard methyl gamma linolenate. Fraction no 6–17 was found to be as methyl gamma linolenate and was pooled together. The percentage purity of isolated methyl gamma linolenate was determined using HPTLC (Fig. 1) and it was confirmed by mass spectral analysis (Fig. 2), ^1^H NMR (Fig. 3 and ^13^C NMR (Fig. 4) analysis. The chemical structure and the flash chromatography report for the separation of GLA-ME are given in Fig. 5 and Fig. 6 respectively.

### Characterization of GLA-ME

FAB-MS: 292 (M, 100 %): 254.70 [^+^(CH_2_)_4_CH = CHCH_2_CH = CH (CH_2_)_4_COOCH_3_] *m/z* + 1, 178.82 [CH_3_(CH_2_)_4_CH = CHCH_2_CH = CHCH_2_CH = CH^+^],151.69 [CH_3_(CH_2_)_4_CH = CHCH_2_CH = CHCH_2_CH^+^] *m/z* + 1, 121.90 [CH3(CH2)4CH = CHCH2CH^+^] *m/z*-3. .

^1^H NMR (300 Mhz,CDCl_3_ δ_H_); 5.5 (m, 6H, olefinic C_6_, C_7_, C_9_,C_10_,C_12_,C_13_), 3.7 (s, 3H, CH_3_ ester C_19_), 2.6 (t, 4H, CH_2_ between double bonds C_8_,C_11_), 2.3 (t, 2H, CH_2_ adjacent to C = O, C_17_), 2.1 (m, 4H, CH_2_ adjacent to double bond C_5_,C_14_), 1.7 (m, 8H, C_3_, C_4_, C_15_ and C_16_) 1.3 (m, 2H, CH_2_ next to CH_3_ C_2_), 0.9 (t, 3H, CH_3_ terminal C_1_) ppm

^13^CNMR (300MHZ,CDCl_3_ δ_C_): 14.14- C_1_, 22.53- C_2_, 24.55 – C_8_, 24.75 - C_11_, 25.60 – C_16_, 26.85 – C_5_, 27.18 - C_14_, 29.21- C_15_, 29.23 – C_4_, 31.72- C_17_, 33.83- C_3_, 51.51- C_19_, 127.56 – C_7_, C_12_, 129.55 – C_9_, C_10_, 130.41 – C_6_, C_13_, 174.82 – C_18_.

### Biological screening

#### Cell lines and culture medium

A-549 cell cultures used in the experiments were procured from National Centre for Cell Sciences, Pune, India. A-549 cells were grown in Earl’s Minimal Essential Medium supplemented with 2 mmol L-glutamine, 10 % Fetal Bovine Serum, Penicillin (100 μg/ml), streptomycin (100 μg/ml) and amphotericin B (5 μg/ml) and the cells were maintained at 37 °C in a humidified atmosphere with 5 % CO_2_ and subculture twice a week.

#### *In-vitro* apoptosis inducing effect

The total cell protein content was determined by sulphoradamine B (SRB) assay [19]. The monolayer cell culture was trypsinized and the cell count adjusted to 1.0 x 10^5^ cell/ml using medium (MEM) containing 10 % newborn calf serum. To each well of the 96 well microtitre plate, 0.1 ml of diluted cell suspension (approximately 10,000 cells) was added. After 24 h, when a partial monolayer was formed, the supernatant was flicked off, the monolayer was washed once and 100 μL of the medium and the culture was exposed to different concentrations of drugs (Table no.1) in microtitre plates. The plates were then incubated at 37 °C for 3 days in 5 % CO_2_ atmosphere, and microscopic examination was carried out and observations recorded every 24 h. After 72 h, 25 μL of 50 % trichloro acetic acid was added to the wells gently such that it forms a thin layer over the drug solution to give an overall concentration of 10 %. The plates were incubated at 4 °C for one hour. The culture plates were flicked and washed five times with tap water to remove traces of medium, drug and serum, and were then air-dried. The air-dried plates were stained with SRB for 30 min. The unbound dye was then removed by rapidly washing four times with 1 % acetic acid. The plates were then air-dried.100 μL of 10 mM Tris base was then added to the wells to solubilise the dye. The plates were shaken vigorously for 5 min. The absorbance was measured using the micro plate reader at a wavelength of 540 nm [20]. The percentage growth inhibition was calculated using the formula below.

$$ \%\ \mathrm{Growth}\ \mathrm{inhibition} = \kern0.75em \frac{\mathrm{Mean}\ \mathrm{O}\mathrm{D}\ \mathrm{of}\ \mathrm{individual}\ \mathrm{test}\ \mathrm{group}}{\mathrm{Mean}\ \mathrm{O}\mathrm{D}\ \mathrm{of}\ \mathrm{control}\ \mathrm{group}}\times 100 $$

## Results and discussion

**Fig. 7:**
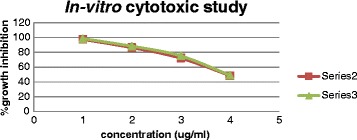
*In-vitro* cytotoxic studies □ GLA-ME, Δ- Standard Rutin

The fatty acid methyl esters (FAME) were prepared from the freeze dried biomass of *Spirulina platensis.* The FAME fraction was subjected to urea fractionation for gamma linolenic acid methyl ester by extraction with n-hexane. This hexane fraction was named as enriched GLA-ME fraction. It was quantified by using HPTLC and was found to contain 57.62 % w/w of GLA-ME. HPTLC method has been developed first time, since already developed methods like gas Chromatography and reverse phase HPLC are tedious and expensive compared to HPTLC which has been proven equal to GC and reverse phase HPLC statistically. The fraction was then directly applied to flash chromatography using hexane: acetone (60:40) as mobile phase. Methyl gamma linolenate was isolated as viscous pale yellow oil (yield 71 % w/v). The spectral analysis. such as mass spectra, ^1^H NMR and ^13^C NMR were consistent with the assigned structure of GLA-ME. The compound was screened for cytotoxicity activity against A-549 cells by determination of CTC_50_ (concentration of the sample required to kill 50 % of the cells) value by SRB assay. The experiments, each were carried out each in triplicate and the results are tabulated in Table 1 and compared with the standard drug Rutin (Fig. 7), a bioflavanol which is a well established promising anticancer agent and its mechanism may be due to the induction of apoptosis [21]. The compound is found to possess comparable cytotoxicity as the standard. However, further detailed investigations on *in-vivo* studies are needed to explore its potential.

**Table 1:** Determination of cytotoxicity by SRB method

S. No.	Compound	Concentration (μM	% Growth Inhibition	CTC_50_
1	GLA ME	3.333	97.45	0.468
2		1.666	86.39	
3		0.833	72.38	
4		0.416	48.45	
5	RUTIN	3.333	98.65	0.442
6		1.666	88.41	
7		0.833	75.25	
8		0.416	49.05	

CTC_50_-concentration of the sample required to kill 50 % of the cell

The novel method has been developed for the isolation of GLA-ME by using a flash chromatography system which improved the percentage yield up to three folds (71 %) in comparison with the conventional methods (18-20 %). The cytotoxicity shown by GLA-ME may be due to the induction of apoptosis of tumor cells by augmenting free radical generation only in the tumor cells but not normal cells. It is evidenced by the research work carried out by Undurti *et al.* [22]. They also reported that induction of apoptosis of tumor cells by GLA is due to its action at the gene/oncogene level and by altering *BCl-2* expression. Hence, it may be concluded the cytotoxicity shown by GLA-ME may be due to induction of apoptosis effect. However, a detailed study of this mechanism is in progress.

## Conclusion

Methyl gamma linolenate was isolated from the *Spirulina platensis* by flash chromatography system and the percentage yield is found to be 71 %. It may be concluded that the Flash chromatography system plays a major role in improving the yield for the isolation of methyl gamma linoleate from *Spirulina platensis*. Since the process consumes very less time compared to conventional isolation, many chemists searching for new technologies for the production of plant constituents of medicinal interest can adopt this method. The isolated molecule is a potent cytotoxic agent towards human lung carcinoma cell lines, it may be further taken up for extensive study.

## Abbreviations

GLA: Gamma linolenic acid
FAME: Fatty acid methyl esters
GLA-ME: Methyl, gamma linolenate

## References

[1] Jubie S, Patil NR, Dhanabal SP, Kalirajan R, Muruganandam N, Shanish A (2012). Synthesis, antidepressant and antimicrobial activities of some novel stearic acid analogues. Eur J Med Chem.

[2] Fedekar FM, Abde WK, Hoda SN (2012). Production and nutritive value of *Spirulina platensis* in reduced cost media. Egypt J Agric Res.

[3] Sajilata MG, Singhal RS, Kamat MY (2008). Fractionation of lipids and purification of g-linolenic acid (GLA) from *Spirulina platensis*. Food Chem.

[4] Martinez JC, Madrid PC, Guerrero JL (2004). Gamma linolenic enrichment from *Borago officinalis* and *Echium fastuosum* seed oils and fatty acids by low temperature crystallization. J Biosci Bioeng.

[5] Spurvey AA, Shahidi F (2000). Concentration of gamma linolenic acid (GLA) from borage oil by urea complexation; Optimization of reaction conditions. J Food Lipids.

[6] Madrid PC, Gurrero JL (2002). High-performance liquid chromatographic purification of gamma linolenic acid (GLA) from the seed oil of two Boraginaceae species. Chromatographia.

[7] Guerrero JL, Madrid PC, Juarez RN (2003). Isolation of some PUFA from edible oils by argentated silica gel chromatography. Grasasy y Aceties.

[8] Jubie S, Dhanabal SP, Afzal Azam M, Muruganantham N, Kalirajan R, Elango K (2013). Synthesis and characterization of some novel fatty acid analogues: A preliminary investigation on their activity against human lung carcinoma cell line. Lipids Health Dis.

[9] Jemal A, Siegel R, Ward E, Hao Y, Xu J, Murray T, Thun MJ (2008). Cancer statistics, 2008. CA Cancer J Clin.

[10] Leary A, Bell N, Darlison L, Guerin M (2008). An analysis of lung cancer clinical nurse specialist workload and value. Cancer Nurs Pract.

[11] Kelly K, Huang C (2008). Biological agents in non-small cell lung cancer: a review of recent advances and clinical results with a focus on epidermal growth factor and vascular endothelial growth factor. J Thorac Oncol.

[12] Mariotto AB, Yabroff KR, Shao Y, Feuer EJ, Brown ML (2011). Projections of the cost of cancer care in the United States: 2010–2020. J Natl Cancer Inst.

[13] Liu B, Cui C, Duan W, Zhao M, Peng S, Wang L, Liu H, Cui G (2009). Synthesis and evaluation of antitumour activities of N4 fatty acyl amino acid derivatives of 1-β-arabinofuranosylcytosine. Eur J Med Chem.

[14] Chhikara BS, Mandal D, Parang K (2010). Synthesis and evaluation of fatty acyl ester derivatives of cytarabine as anti-leukemia agents. Eur J Med Chem.

[15] Kenny FS, Pinder SE, Ellis IO, Gee JM, Nicholson RI, Bryce RP, Robertson JF (2000). Gamma linolenic acid with tamoxifen as primary therapy in breast cancer. Int J Cancer.

[16] Bunce OR, Abou-El Ela SH (1990). Eicosanoid synthesis and ornithine decarboxylase activity in mammary tumors of rats fed varying levels and types of N-3 and/or N-6 fatty acids. Prostaglandine Leukot Essent Fatty Acids.

[17] Menendez JA, Del Mar BM, Montero S, Sevilla E, Escrich E, Solanas M, Cortes-Funes H, Colomer R (2001). Effects of gamma-linolenic acid and oleic acid on paclitaxel cytotoxicity in human breast cancer cells. Eur J Cancer.

[18] Plumb JA, Luo W, Kerr DJ (1993). Effect of polyunsaturated fatty acids on the drug sensitivity of human tumour cell lines resistant to either cisplatin or doxorubicin. Br J Cancer.

[19] Jubie S, Gayathri R, Srividya AR, Kalirajan R, Prabitha P, Sankar S, Elango K (2011). Synthesis and characterization of some novel quinoxaline-2, 3-dione derivatives: a preliminary investigation on their activity against a human epithelial carcinoma cell line. Lett Drug Design Discov.

[20] Vijayan P, Vinod Kumar S, Dhanaraj SA, Mukhergee PK, Suresh B (2003). *In vitro* cytotoxicity and antitumour properties of *Hypericum mysorense* and *Hypericum patulum*. Phytother Res.

[21] Ren W, Qiao Z, Wang H, Zhu L, Zhang L (2003). Flavanoids : Promising Anticancer Agents. Med Res Rev.

[22] Undurti ND (2006). Tumoricidal and anti-angiogenic actions of gamma-linolenia acid and its derivatives. Curr Pharm Biotechnol.

